# An In Vivo Biostability Evaluation of ALD and Parylene‐ALD Multilayers as Micro‐Packaging Solutions for Small Single‐Chip Implants

**DOI:** 10.1002/smll.202410141

**Published:** 2025-01-23

**Authors:** Kambiz Nanbakhsh, Matthias Van Gompel, Riina Ritasalo, Astrid Gollhardt, Domonkos Horváth, Kinga Tóth, Domokos Meszéna, István Ulbert, Wouter Serdijn, Vasiliki Giagka

**Affiliations:** ^1^ Department of Microelectronics Faculty of Electrical Engineering Mathematics and Computer Science Delft University of Technology Delft 2628 CN The Netherlands; ^2^ Comelec SA La Chaux‐de‐Fonds 2301 Switzerland; ^3^ Applied Materials, Finland Masalantie 365 Masala 02430 Finland; ^4^ Department of Environmental & Reliability Engineering Fraunhofer Institute for Reliability and Microintegration IZM 13355 Berlin Germany; ^5^ Research Centre for Natural Sciences Institute of Cognitive Neuroscience and Psychology HUN‐REN Budapest 1117 Hungary; ^6^ Faculty of Information Technology and Bionics Pazmany Peter Catholic University Budapest 1083 Hungary; ^7^ Department of Neurosurgery and Neurointervention Faculty of Medicine Semmelweis University Amerikai út 57 Budapest 1145 Hungary; ^8^ Department of Neuroscience Erasmus Medical Center Rotterdam 3015 GD The Netherlands; ^9^ Department of System Integration and Interconnection Technologies Fraunhofer Institute for Reliability and Microintegration IZM 13355 Berlin Germany

**Keywords:** atomic layer deposition, biofluid barriers, micro‐packaging, Parylene, PDMS

## Abstract

Miniaturization of next‐generation active neural implants requires novel micro‐packaging solutions that can maintain their long‐term coating performance in the body. This work presents two thin‐film coatings and evaluates their biostability and in vivo performance over a 7‐month animal study. To evaluate the coatings on representative surfaces, two silicon microchips with different surface microtopography are used. Microchips are coated with either a ≈100 nm thick inorganic hafnium‐based multilayer deposited via atomic layer deposition (ALD‐ML), or a ≈6 µm thick hybrid organic–inorganic Parylene C and titanium‐based ALD multilayer stack (ParC‐ALD‐ML). After 7 months of direct exposure to the body environment, the multilayer coatings are evaluated using optical and cross‐sectional scanning electron microscopy. Time‐of‐flight secondary ion mass spectrometry (ToF‐SIMS) is also used to evaluate the chemical stability and barrier performance of the layers after long‐term exposure to body media. Results showed the excellent biostability of the 100 nm ALD‐ML coating with no ionic penetration within the layer. For the ParC‐ALD‐ML, concurrent surface degradation and ion ingress are detected within the top ≈70 nm of the outer Parylene C layer. The results and evaluation techniques presented here can enable future material selection, packaging, and analysis, enhancing the functional stability of future chip‐embedded neural implants.

## Graphical abstract

This study investigates thin‐film stacks combining organic and inorganic materials as conformal packaging coatings for CMOS microchips in next‐generation neural implants. Using advanced material analysis, two coatings—an inorganic 100 nm hafnium‐based ALD multilayer and a hybrid ParC‐titanium‐based ALD stack—were evaluated after a 7‐month in vivo animal study. Results demonstrate excellent biostability and barrier properties, offering critical insights for future neural implant designs.

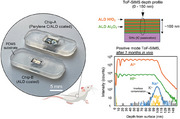

## 1 Introduction

Next‐generation single‐chip neural implants are emerging with the potential to revolutionize the field of neuroscience and healthcare.^[^ ^1^, ^2^, ^3^, ^4^, ^5^ ^]^ Packaging of these miniaturized devices, however, has been identified as one of the major challenges as biocompatibility, biostability, and effective device protection should be validated for the packaging material through long‐term in vivo studies.^[^ ^6^, ^7^, ^8^, ^9^, ^10^ ^]^ In the last years, various organic and inorganic thin‐film coatings have been investigated as chip‐scale packaging solutions for implantable devices.^[^ ^2^, ^11^, ^12^, ^13^ ^]^ Inorganic thin‐film ceramics such as SiN_X_, SiO_X_, SiC, HfO_2_ and Al_2_O_3_ can be deposited using thermal, various plasma‐enhanced chemical vapor deposition (PECVD) or atomic layer deposition (ALD) processes.^[^ ^14^, ^15^ ^]^ These inorganic films can yield low water vapor transmission rates (WVTR) (as low as 10^−6^ g cm^−2^ day^−1^) while having thicknesses ranging from 10 nm to 1 µm.^[^ ^13^, ^15^, ^16^, ^17^, ^18^ ^]^ The downside of these inorganic films, however, is their brittleness and potential to crack in case of mechanical stress. To assess the mechanical integrity of these films, parameters such as crack onset strain^[^ ^19^ ^]^ (the strain at which cracking begins) and fracture toughness^[^ ^20^ ^]^ (resistance to crack propagation) are commonly used. For instance, HfO_2_​, SiN_X,_ and Al_2_O_3_ films can typically have crack onset strain values ranging from 0.5% to 2%, depending on the deposition method and film thickness.^[^ ^21^ ^]^

Comparatively, organic films like Parylene C have higher WVTR (≈10^−1^ g cm^−2^ day^−1^) but are more mechanically flexible and less susceptible to cracking. Multilayered hybrid organic–inorganic thin‐film coatings have been demonstrated to have higher barrier properties compared to single organic layers as they create a meandering path for diffusing moisture and ions^[^ ^24^ ^]^ resulting in a lower WVTR and better flexibility. These films are created by sandwiching the ceramic barrier layers (deposited using either ALD or PECVD) between organic polymer layers like Parylene^[^ ^19^, ^22^ ^]^ or polyimide.^[^ ^23^ ^]^ Stacking more layers, however, does not necessarily increase the protection performance of the multilayer coating. As the hybrid coating includes multiple organic‐ceramic interfaces, each interface is susceptible to delamination if adhesion is not optimized, potentially compromising the coating's performance.^[^ ^24^ ^]^ Moreso, throughout the lifetime operation of the device, aging of the materials in the body (chemically and mechanically), can weaken the interfaces and lead to warping and delamination.^[^ ^22^, ^25^ ^]^ Similarly, the surface topography of the substrate (device to be coated) can also introduce stresses in the coating stack, weakening its mechanical integrity.^[^ ^25^ ^]^ It has also been shown that poor adhesion between the applied coating and the substrate can result in delamination and bulging of the coating.^[^ ^26^ ^]^ Therefore, besides the WVTR, engineering and optimizing the coating stack according to the substrate is critical in ensuring successful, long‐lasting protection.

As the devices are getting smaller and the coating layers thinner, the requirements for a conformal and defect‐free coating are getting tighter. At the same time, evaluation of these coatings is also getting more challenging, with new and more sensitive techniques being regularly proposed.^[^ ^27^, ^28^, ^29^, ^30^, ^31^ ^]^ Among these techniques, magnesium and calcium tests are perhaps some of the most sensitive methods for evaluating the moisture barrier properties of thin‐film coatings.^[^ ^32^, ^33^ ^]^ While a method for in situ encapsulation monitoring using magnesium testing was proposed and validated in vivo, these techniques, are usually conducted in vitro and on non‐representative material surfaces with flat features. As a result, they fall short in considering the biodegradation mechanisms experienced by materials in vivo, as well as the interaction of the substrate material and its topography on the overall coating performance.

As materials are exposed to the in vivo environment, gradual material degradation can be expected which can lower their performance and barrier properties. Previous investigations have demonstrated the degrading effect of the body environment on various ceramic and polymer materials.^[^ ^34^, ^35^, ^36^, ^37^ ^]^ Understanding the in vivo behavior of these novel thin‐film coatings can help engineer high‐performance, long‐lasting materials and extend their practical applications in micro‐packaging for implantable ICs.

**Figure 1:**
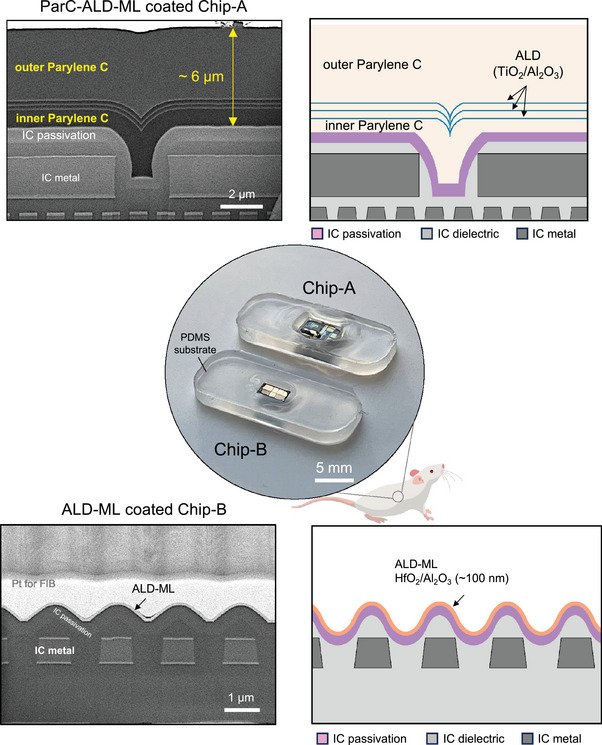
Optical and cross‐sectional scanning electron microscope (SEM) images of two CMOS microchips (Chip‐A and Chip‐B) with different surface microtopography coated with ParC‐ALD‐ML and ALD‐ML thin‐film coatings. Top cross‐sectional SEM image and schematic showing the ParC‐ALD‐ML coating deposited on Chip‐A with a total thickness of ≈6 µm, conformally coating the chip surface. Bottom cross‐sectional SEM image and schematic showing the ALD‐ML applied on Chip‐B with a total thickness of ≈100 nm. Coated microchips are placed on a PDMS substrate which is used as a handling carrier (center image). The top surface of all coated chips is directly exposed to the in vivo environment.

In this work, we evaluated the long‐term in vivo performance and biostability of two thin‐film coatings: an organic–inorganic Parylene C titanium‐based ALD multilayer film, from here on referred to as ParC‐ALD‐ML (≈6 µm in thickness), and an inorganic HfO_2_/Al_2_O_3_ ALD multilayer from here on referred to ALD‐ML (≈100 nm in thickness) (see **Figure** **1** and Experimental Section). To evaluate the coating films on representative surface materials, the coatings were deposited on two different silicon microchips (*n* = 12 chips in total) fabricated by two different commercial complementary metal‐oxide semiconductor (CMOS) foundries, from here on referred to as Chip‐A and Chip‐B (see Figure 1 and Experimental Section). Each microchip exhibited different surface microtopography, influenced by the use of the top metallization of the CMOS process (see Figure S1, Supporting Information). Such microtopography can even compromise the conformality of the IC's own SiN_X_/SiO_X_ passivation layers, creating easy ingress points for corrosive body fluids within the chip.^[^ ^38^ ^]^ For both chips, the passivation layer (the final foundry deposited coating layer) covering the top side of the chip was SiN_X_ with aluminum (Al) used for the top metallization and the bond pads. In our previous investigation,^[^ ^38^ ^]^ by soaking uncoated microchips in 67 °C phosphate buffered saline solution and routine electrical monitoring, the main degradation pathways for chip failure were identified to be: 1) the dissolution of the top SiN_X_ passivation, specially near the edges of the top metal, and, 2) IC pad corrosion.

The ParC‐ALD‐ML stack was optimized to create a conformal coating on the microtopography of the chips (Figure 1a). The stack consisted of an ≈6 µm organic–inorganic structure, comprising an inner 1 µm Parylene C layer, three ALD/Parylene C dyads, with each ALD layer being a TiO_2_/Al_2_O_3_ bilayer ≈30 nm thick and the Parylene C layer being ≈250 nm thick, and a final top 4 µm outer Parylene C film. Depositions of the materials were done consecutively in the same vacuum vessel and without venting cycles, eliminating any contamination between the polymer and metal‐oxide ALD layers. As Parylene has been shown to have poor adhesion to silicon‐based ceramics,^[^ ^26^, ^39^ ^]^ for adhesion optimization, in situ surface activation followed by a seed Al_2_O_3_ ALD (<10 nm) deposition was done prior to the inner Parylene C layer.

For the ALD‐ML, we used x10 layers of HfO_2_ (10nm)/Al_2_O_3_ (10nm). Al_2_O_3_ ALD layers have been reported to have one of the highest water vapor barrier properties,^[^ ^14^, ^15^ ^]^ however, their weak chemical stability in moist environments can gradually jeopardize their barrier properties.^[^ ^40^ ^]^ For this purpose, we chose to have the HfO_2_ as the final capping layer, which has shown to be highly stable when soaked in vitro in phosphate buffered saline (PBS) solution.^[^ ^11^, ^39^ ^]^

The two investigated thin‐film coatings were applied on the microchips using the presented processes given in the Experimental Section. Coated chips were placed on biocompatible PDMS substrates (NuSil MED2‐4213) to ease handling and implantation (Figure 1). The edges of the coated microchips were also covered with the soft PDMS. The top surface, however, was mainly left uncoated, directly exposing the ParC‐ALD‐ML and ALD‐ML films to tissue. Samples were then subcutaneously implanted in rats. After 2, 4, and 7 months of in vivo aging, explanted microchips were evaluated using cross‐sectional scanning electron microscopy (SEM) and atomic force microscopy (AFM), while the chemical stability and barrier properties of the coatings were analyzed using time‐of‐flight secondary ion mass spectrometry (ToF‐SIMS) (See Table S1, Supporting Information).

## 2 Results

### 2.1 In Vivo Structural Stability of Multilayer Coatings

**Figure 2:**
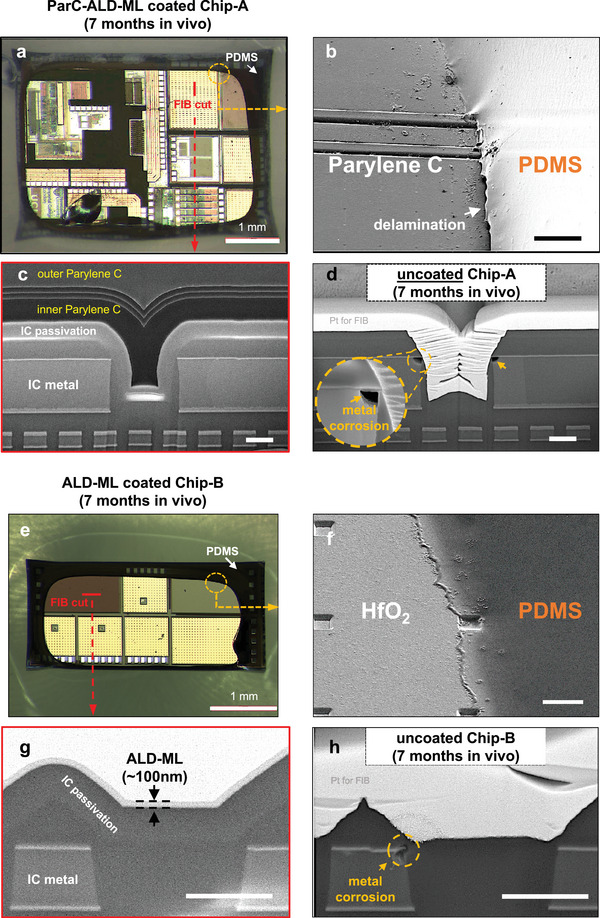
Optical micrograph and SEM images of the Par‐ALD‐ML and ALD‐ML coatings after 7 months in vivo implantation. a) Optical micrograph showing a Chip‐A coated with ParC‐ALD‐ML with no visible signs of degradation on the IC. b) Titled SEM image near the PDMS‐coated region with interfacial delamination between PDMS and Parylene. Scale bar is 100 µm. c) Cross‐sectional SEM images showing the ParC‐ALD‐ML stack after implantation. d) Cross‐sectional SEM image of a similar Chip‐A with no coating after 7 month body exposure showing dissolution of the IC passivation and metal corrosion. Scale bar for (c) and (d) is 1 µm. e) Optical micrograph of a representative Chip‐B coated with ALD‐ML with no visible signs of degradation. f) Tilted SEM image near PDMS‐coated region with no signs of PDMS delamination. g) Cross‐sectional SEM image showing intact ALD‐ML and IC passivation and metals. h) Cross‐sectional SEM image of a similar Chip‐B with no coating showing corrosion of IC. Scale bar for (f), (g), and (h) is 1 µm.

To evaluate the structural stability of the multilayer coatings, all explanted microchips were examined using optical and electron microscopy. **Figure** **2** shows two representative microchips encapsulated with a ParC‐ALD‐ML and ALD‐ML coating, explanted after 7 months of implantation in rats. For these representative samples, ParC‐ALD‐ML was used for encapsulating Chip‐A (Figure 2a–d), and the 100 nm ALD‐ML was used for encapsulating a Chip‐B IC (Figure 2e–h).

For both microchips, light microscopy (up to 100x magnification) showed no visible signs of degradation on the coating or IC surface across the entire chip area. Additionally, the aluminum IC pads coated using the thin layers showed no signs of metal corrosion. Microscopic results, however, revealed significant delamination between the PDMS and the Parylene C surface, allowing lateral ingress of body fluids under the PDMS (Figure 2b). Delamination of the PDMS‐Parylene interface was noticed for the 2‐month explanted microchips and was observed on all ParC‐ALD‐ML coated chips (*n* = 6), indicating the poor in vivo stability of this interface. All microchips encapsulated with the ALD‐ML (*n* = 6), on the other hand, showed stability at the PDMS‐HfO_2_ interface with no signs of delamination when examining the PDMS coated edges of the chips (see Figure 2f).

The structural stability of the multilayer coating stacks was analyzed using cross‐sectional SEM imaging. Focused ion beam (FIB) cross‐sections were made on at least three locations of each explanted microchip. Figure 2c shows a representative SEM cross‐section of the ParC‐ALD‐ML stack on Chip‐B after 7 months of implantation, demonstrating the stability of the entire multilayer stack. SEM cross‐section results also indicate the adhesion stability between the ParC‐ALD‐ML coating and the IC's passivation surface, even on the most complex microtopography on the chip (Figure 2c). For comparison, Figure 2d shows an uncoated Chip‐A IC after 7 months of implantation. Comparing Figure 2c,d demonstrates how the ParC‐ALD‐ML coating protected the IC's passivation and metallization from any degradation and corrosion.

Figure 2e demonstrates a cross‐sectional SEM image of the ALD‐ML coating on a Chip‐B sample after 7 months of implantation. With regards to the nanolaminate film thickness, no variation was observed after the in vivo study (≈100 nm). Similar cross‐section SEM investigations on other locations of the chip also showed the expected thickness of the ALD‐ML with no delamination from the chip surface.

For comparison, Figure 2h shows an uncoated Chip‐B IC after 7months of implantation in rat where degradation of the IC's passivation and metal corrosion is seen. Comparing the ALD‐coated and uncoated ICs shows how a 100 nm ALD‐ML coating can protect the chip from degradation.

SEM surface analysis was done on the 4 and 7‐month explanted chips to detect any defects, openings or degradation of the coating layers. As an example, Figure S1 (Supporting Information) shows a 7‐month explanted ALD‐ML coated Chip‐B with openings in the coating layer. The size of the largest opening is measured to be ≈5 µm x 10 µm. Cross‐sectional SEM evaluations in the center of the opening showed the degradation of the IC's silicon nitride (SiN_X_) passivation layer. Through the opening in the ALD‐ML coating, the chip's SiN_X_ layer was exposed to the body environment (ionic fluids, proteins, enzymes), causing a ≈400 nm dissolution of the microchip's SiN_X_ layer. Other analyzed ALD‐ML coated chips did not reveal any defects or openings. Although ALD coatings are known for their high conformality and defect‐free nature, such an opening may result from the presence of a surface particle during the coating process, which could dislodge post‐coating and leave the underlying area exposed. In contrast, ParC‐ALD‐ML coatings applied on four microchips which were explanted at 4 (*n* = 2) and 7 (*n* = 2) months exhibited no defects or openings. This could be due to the fact that the thicker ParC‐ALD‐ML coating can coat surface particles and prevent them from being displaced (see Figure S4, Supporting Information).

**Figure 3:**
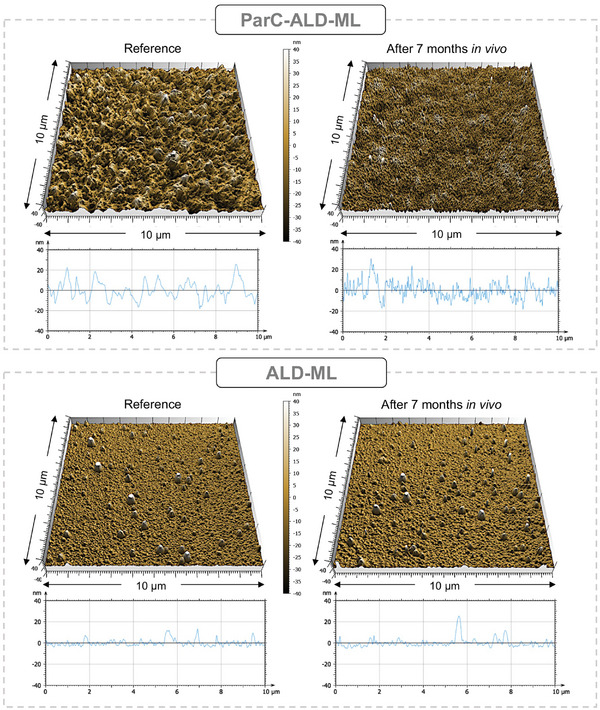
Atomic force microscopy (AFM) comparing the outer surface roughness of the multilayer coatings after 7 months in vivo implantation with a reference (non‐implanted) sample. Top graphs comparing the Parylene C surface showing nano roughening appearing on the surface after implantation. Bottom graphs show no change in surface roughness for the HfO_2_ ALD layer after long‐term implantation.

The surface stability of the coating layers was examined using AFM. **Figure** **3** presents the AFM results for representative ParC‐ALD‐ML and ALD‐ML coated chips at 7 months in comparison to pristine un‐implanted samples. Comparing the surface topography of the ParC‐ALD‐ML shows that exposure to the body environment results in slight nano‐roughening of the surface Parylene C layer. The ALD‐ML, however, showed no changes in its surface topography.

### 2.2 In Vivo Chemical Stability and Barrier Properties

The chemical stability and barrier properties of both coating layers were examined using ToF‐SIMS depth profile analysis. ToF‐SIMS depth profiles were collected at negative and positive modes, and analyzed over a 50 × 50 µm^2^ area at two different locations on each microchip. Negative mode depth profiling was used to evaluate the oxygen and moisture ingress levels by monitoring the [O^−^] and [OH^−^] cluster ions in the layers. In addition, the ingress of various anions found in body fluids (Cl^−^, PO_4_^−^, and S^−^) was evaluated using negative mode analysis. Positive mode depth profiling was used to detect traces of penetrating cations (Na^+^, K^+^, Ca^+^, and Mg^+^).

**Figure 4:**
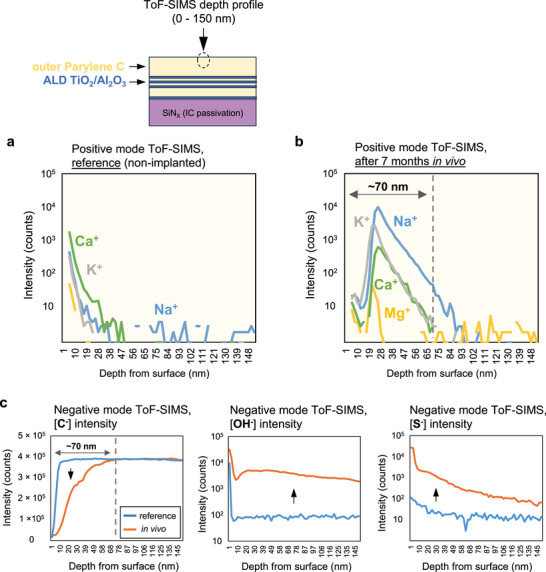
Positive and negative mode ToF‐SIMS depth profiles of ParC‐ALD‐ML coatings, comparing reference (non‐implanted) with 7‐month explanted devices. a,b) Positive mode depth profiles from 0–150 nm for (a) reference (non‐implanted), and (b) implanted, showing ionic penetration within the ≈70 nm outer Parylene C layer. c) Negative mode depth profiles from 0–150 nm comparing the [C^−^], [OH^−^], and [S^−^] intensities on reference (non‐implanted) and 7‐month explanted devices. Lower carbon intensity for the first ≈70 nm Parylene C suggests degradation of the polymer.

**Figure** **4** compares the depth profiles of reference (non‐implanted) and implanted ParC‐ALD‐ML coatings on two separate microchips. Depth profiles were collected from surface (0 nm) to a ≈150 nm depth, analyzing the outer Parylene C layer of the multilayer stack that is exposed to tissue. Positive mode ToF‐SIMS depth profile results showed peaks of Ca^+^, Na^+^, and K^+^ cations around a depth of about ≈20 nm, suggesting penetration of these ions. The ionic intensities decay and reach the instrument detection limit as the profile reaches a depth of ≈70 nm within the layer. The reference Parylene C sample, however, did not show any ionic impurities in the layer.

To evaluate the chemical stability of the Parylene C layer, ToF‐SIMS depth profiling was done in negative mode to monitor the carbon [C^−^] intensity in the layer. In the first ≈70 nm, a drop in the carbon intensities is observed compared to the bulk material and, to the reference sample. Other carbon related cluster ions specific for Parylene C (such as [CCl^−^]) also showed a drop in the first 70 nm of the implanted material. This reduction in carbon intensity suggests potential degradation of the Parylene C layer after prolonged exposure to the body environment. Results in negative mode also indicated higher intensities of [OH^−^] and [S^−^] for the 7‐month implanted Parylene layer as compared to the reference layer. The higher [OH^−^] could be due to the gradual moisture penetration within the layer.

The detected sulfur ions [S^−^] likely originate from sulfur‐containing compounds in the tissue or body fluids, gradually infiltrating the layer.^[^ ^41^, ^42^ ^]^ However, the [S^−^] intensity decreases beyond a depth of 100 nm, eventually matching the levels observed in the reference sample. The reduced carbon intensity and the presence of ionic ingress within the first ≈70 nm of the Parylene C layer could suggest concurrent surface degradation and ionic penetration following the long‐term exposure of the polymer to body environment.

**Figure 5:**
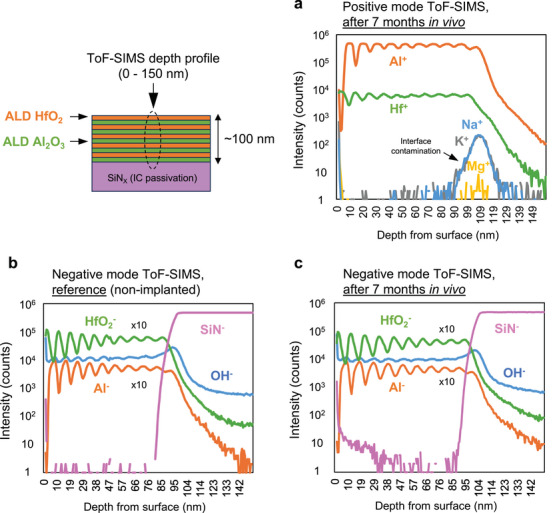
Positive and negative ToF‐SIMS depth profiles of the ALD‐ML coating comparing reference (non‐implanted) and 7‐month implanted devices. a) Positive mode depth profile from 0–150 nm showing no ion penetration in the HfO_2_–Al_2_O_3_ multilayer. b,c) Negative mode depth profiles from 0–150 nm comparing the [Al^−^], [HfO_2_^−^], and [OH^−^] intensities for a (b) reference (non‐implanted) and a (c) 7‐month explanted device showing chemical stability of the entire ALD‐ML stack.

Negative and positive mode ToF‐SIMS depth profiles of a representative ALD‐ML coating after 7 months in vivo exposure appears in **Figure** **5** and is compared to a reference (non‐implanted) sample. Positive mode depth profiles (Figure 5a) show no indication of cation ingress (Ca^+^, Mg^+^, Na^+^, and K^+^) in the layer. The peak observed at ≈109 nm is due to surface contamination, already present before coating. Reference samples also showed a similar peak at the ALD‐chip interface. The ALD‐ML stack consists of 10 HfO_2_ layers and 10 Al_2_O_3_ layers with the HfO_2_ layer at the surface. Both positive and negative depth profiles show the presence of all 10 peaks for the 7‐month explanted sample. Comparing the intensity between the reference and explanted samples (Figure 5b,c) shows no change in the intensity of the profiles demonstrating the outstanding in vivo stability of the thin ceramic layers. Additionally, the [OH^−^] signal also remains stable and similar to the reference sample indicating the high moisture barrier properties of the hafnia‐based ALD layer.

## 3 Discussions and Conclusion

**Table** **1** summarizes and compares the findings for the two thin‐film multilayer coatings after the 7‐month in vivo study. Both films were engineered to have conformality to the ICs topography (aspect rations: 3 µm (height)/1 µm (width) for Chip‐A and 0.9 µm (height)/2 µm (width) for Chip‐B, see Figure S5, Supporting Information) and maintain their internal structural stability through the in vivo study. The coatings were found to effectively protect the aluminum pads and SiN_X_ passivation on the IC from any biodegradation. Out of the tested ALD‐ML coated ICs (*n* = 6 in total) no PDMS‐ALD delamination was observed even when applying a shear force to the PDMS to qualitatively evaluate its adhesion (see Note S1, Supporting Information). This suggests the excellent in vivo adhesion of PDMS to ALD HfO_2_. More significantly, the top 10 nm HfO_2_ layer exposed to tissue showed superior stability as no dissolution or hydration was observed which has been reported for other ceramics barriers like SiC, SiO_X_, and SiN_X_.^[^ ^38^, ^43^, ^44^ ^]^ In combination, these insights make HfO_2_‐based ALDs a very promising bioceramic for chronic applications.

**Table 1:** Summary of the 7‐month in vivo study.

Coating	Conformality on IC	IC pad protection	IC passivation protection	PDMS adhesion	Chemical stability	Ionic barrier properties
ALD‐ML (≈100nm)	High	High	High	High	No degradation	High
ParC‐ALD‐ML (≈6 µm)	High	High	High	Low	Top ≈70nm degradation	Ion ingress in top ≈70 nm

For the ParC‐ALD‐ML, the observed degradation and ion ingress in the top Parylene C layer exposed to tissue suggests concurrent biodegradation and ion penetration. To mitigate this, a biostable ALD ceramic layer can be applied to cap the Parylene layer. Additionally, PDMS could be used as the outer tissue‐contacting layer on top of Parylene C. However, in this case, the poor adhesion between PDMS and Parylene must be enhanced, potentially by inserting a thin ceramic layer in between which can act as an adhesion promoter.^[^ ^45^, ^46^ ^]^

As mentioned in the introduction, for the hybrid layer, due to the presence of multiple organic‐ceramic interfaces, the mechanical integrity of the hybrid stack is of concern. To compare the mechanical integrity of the hybrid Parylene C layer to a single layer, as supporting experiments, a group of ICs were also coated using a single Parylene C layer, ≈4 µm (see Figure S5, Supporting Information). Post‐in vivo aging showed no difference between the single and hybrid coating, demonstrating the mechanical stability of the hybrid multilayer. In terms of aluminum pad and IC passivation protection, the single Parylene C layer also showed no observable degradations, suggesting that for durations <1 year, a single Parylene C layer could perhaps also protect the chip.

In fact, for implantable electronics, one of the primary goals of applying a coating barrier is to mitigate moisture‐induced failures (e.g., corrosion^[^ ^47^ ^]^ hydration/oxidation,^[^ ^38^, ^44^ ^]^ or shunt leakage paths)^[^ ^26^ ^]^ by delaying moisture permeation. Consequently, the selection of a coating based on its WVTR depends on two key factors: 1) the moisture‐sensitivity level of the electronic materials and circuitry underneath, and 2) the intended operational lifetime of the device (see Note S2, Supporting Information). For short‐term applications (1–3 months), if cost is not of concern, using the hybrid layer over a single Parylene C layer could increase device reliability, especially if the moisture‐sensitivity level of the electronic components is unknown. However, when designing systems for long‐term applications (>1 year), a low WVTR is essential to delay moisture‐induced failures.

The tissue reaction around all implanted devices at months 2, 4, and 7 was carefully examined. All samples showed no adhering tissue to the surface of the coatings and could be easily removed in pristine condition from the fibrotic capsules suggesting the biocompatibility of the exposed HfO_2_ and Parylene C layer for the ALD‐ML and ParC‐ALD‐ML coatings, respectively (Figure S2, Supporting Information).

Throughout the 7‐month implantation study, the excellent in vivo biostability and barrier properties of the HfO_2_ ALD were demonstrated. However, due to the thinness of ALD protective coatings, any imperfection (defect or crack) in the layer can allow body fluid penetration in the layer, causing gradual degradation and corrosion of the underlying IC materials. To slow the degradation, the use of a final PDMS coating on the ALD could be explored as it covers the imperfections and prevents direct contact between the body fluids and the layer. Moreover, the demonstrated adhesion of PDMS and HfO_2_ makes this combination stack a suitable long‐lasting packaging solution with PDMS acting as the soft compliant tissue contacting material. In the case of the Par‐ALD‐ML coated chips, no imperfections in the coating were observed when analyzing *n* = 4 coated chips, suggesting that the Par‐ALD‐ML may be used as a standalone coating when exposed to the body. Nevertheless, ToF‐SIMS depth profiling indicated degradation of the outer Parylene C layer within ≈70 nm depths in the layer. Assuming a surface‐to‐bulk Parylene C degradation rate of ≈10 nm month^−1^, it would still take >10 years for the entire 4 µm outer Parylene to degrade. For organic/inorganic multilayer barriers, however, the main criterion for the material stack is to maintain its mechanical integrity throughout the intended lifetime in the body.

For encapsulating flexible implantable electronics comprising both rigid and flexible regions, the results presented here suggest that the two investigated coatings can provide a viable solution for coating rigid structures such as the silicon IC. However, for the flexible regions of the device, the ALD coating is unsuitable, as flexure can readily induce cracks in the brittle coating, significantly compromising its barrier properties. This limitation of ALD coatings has been previously evaluated and quantified, with studies showing a 23‐fold increase in WVTR after bending the substrate with a radius of 5 mm.^[^ ^13^ ^]^ For flexible regions, the hybrid ParC‐ALD‐ML encapsulation is a more suitable alternative. For improving the interface adhesion between the coating and the flex polymer substrate, techniques such as vapor phase infiltration could also be explored to enhance the hermeticity and mechanical integrity of the encapsulation.^[^ ^48^ ^]^

For embedded microsystems,^[^ ^49^, ^50^, ^51^ ^]^ applying the thin‐film coatings proposed here requires detailed attention for a successful and long‐term encapsulation. Component stand‐off and surface cleanliness are two main concerns that should be carefully assessed (see Note S3, Supporting Information). Cleanliness of the surface has a significant effect on the adhesion and encapsulation performance.^[^ ^52^, ^53^ ^]^ For embedded systems that are assembled using surface mount technology and soldering, dedicated steps are needed to ensure a flux‐free surface after assembly. Flux residues can significantly impact the encapsulation performance as they can both impact adhesion and aid in the surface condensation of moisture, expediting the development of shut leakage paths.^[^ ^47^ ^]^

In this investigation, both microchips were designed to incorporate structures with the highest aspect ratios achievable within their respective fabrication processes. For applications using other IC technology nodes, particularly those utilizing the thick top metallization, it is important to consider the aspect ratios during the design stage. The selection and optimization of the thin‐film packaging should be based on these aspect ratios to minimize stress and improve the coating conformality. Additionally, increasing the pitch between IC pads can provide more surface area for encapsulation to adhere to the substrate, thereby reducing the likelihood of shunt leakage paths.

The microchips here were not electrically connected and activated. For electrical connections to the IC, generally, gold (Au) wire‐bonding is used. Gold, however, has been reported to exhibit weak adhesion to polymers which can compromise the integrity of the coating.^[^ ^44^, ^54^ ^]^ Moreover, the gold‐aluminum interface created between the wire and the IC's aluminum pad has been shown to be susceptible to galvanic corrosion for devices packaged with moisture‐permeable polymers.^[^ ^38^, ^47^, ^55^ ^]^ As a future investigation, the effectiveness of the thin‐film coatings can be examined in protecting wire‐bond connections.

## 4 Experimental Section

### Silicon‐IC Microchips

For evaluating the thin‐film coating layers, two different CMOS microchips with different surface microtopography were used as test vehicles. Both chips were fabricated in a standard CMOS foundry: Chip‐A was fabricated in a 4‐metal, 0.35 µm process with a thick top metal (2.8 µm) and had a total area of 4 mm x 5 mm (20 mm^2^). Thick top metals have been used for implementing on‐chip coils for wireless powering and communication^[^ ^11^ ^]^ Chip‐B was fabricated using a 6‐metal, 0.18 µm process with nominal top metal, having a total area of 1.7 mm x 3.5 mm (5.9 mm^2^).

### HfO_2_‐Based ALD Multilayer (ALD‐ML) Coating

A 100 nm HfO_2_‐based ALD stack was deposited using x10 layers of HfO_2_ (10 nm) and Al_2_O_3_(10 nm) at the Picosun Oy company using their Applied^TM^ Picosun^TM^ R‐200^TM^ Advanced ALD reactor, at a pressure of ≈1 mbar (N_2_ atm.). The PicoHot^TM^ source system (PH‐300) and PicoSolution^TM^ (both Picosun Oy) precursors were vaporized from stainless‐steel precursor bottles at increased and room temperature, respectively. The Al_2_O_3_ was deposited first, following alternating layers of HfO_2_ and Al_2_O_3_ with the HfO_2_ layer as the final capping layer. The layers were deposited at a pressure of ≈1 mbar (N_2_ atm.) using thermal ALD‐processes at 200 °C. For the ALD deposition, microchips were placed on mesh grids to allow a conformal coating of the microchips. More data on the deposition process can be found elsewhere.^[^ ^13^ ^]^

For the ALD‐ML, the thickness of each layer was chosen based on the following. For the single layer, the thickness was chosen based on the minimal needed thickness for a complete chemical reaction, budling a complete and closed layer. For the full stack, a ≈100 nm thickness (x10 stacks of each) was chosen to create an ALD coating with the least possible all‐through defects.

### Parylene‐ALD Hybrid Multilayer (ParC‐ALD‐ML) Coating

A ≈6 µm organic–inorganic stack consisting of Parylene C and Al_2_O_3_‐TiO_2_ ALD multilayers were deposited at Comelec using the C30H Parylene‐ALD hybrid deposition system. The stack consists of a 1 µm Parylene C film, three dyads (Al_2_O_3_/TiO_2_/Parylene C), and a final 4 µm top Parylene C layer. Depositions of the materials were done consecutively in the same vacuum vessel and without venting cycles. This eliminates any contamination between the polymer and metal‐oxide ALD layers, usually present after handling substrates. The Parylene C layers were deposited at room temperature (23 °C). Prior to coating, an initial O_2_ plasma treatment step followed by a silane (A174) adhesion promoter step was included in the cycle. Within the multilayer stack, to optimize the interfacial adhesion between the inorganic and organic layers, silane adhesion promoter or a plasma step was used. The metal oxide ALD layers were deposited at temperatures slightly below 100 °C to minimize interfacial stresses caused by the mismatch in the coefficients of thermal expansion (CTE) between Parylene C and the inorganic ALD layers.

For the ParC‐ALD‐ML, the thicknesses were chosen based on the following. The first Parylene C layer was chosen based on the surface step present on the microchips. The goal of the first layer will was to cover the surface asperities of the IC and create a rather smoother surface. The ALD TiO_2_/Al_2_O_3_ ratio was chosen based on three inputs: 1) the minimal needed thickness to have a closed layer, 2) a total thickness needed for a sufficient barrier and, 3) low thickness for mechanical robustness (thicker layers tend to crack). What fulfills all three conditions and minimizes deposition time (and cost) was chosen.

### PDMS Substrate and Partial PDMS Coating

The chips were placed on 3 mm thick, soft PDMS substrates fabricated from medical‐grade silicone rubber (MED2‐4213, NuSil). For manufacturing the PDMS substrates, a custom‐made polytetrafluoroethylene (PTFE) mold was filled with uncured PDMS, which was then semi‐cured in the oven at 100 °C for 30 minutes. At the same time, for optimal PDMS adhesion, the coated ICs were surface activated and cleaned using UV‐ozone for 15 min with their top passivation facing the ozone lamp. The ICs were then placed in the center of the semi‐cured PDMS substrate. After positioning, the surrounding edges of the chips were coated using the same medical grade PDMS (MED2‐4213, NuSil) to smooth the sharp edges of the IC structure. Finally, the samples were fully cured in the oven at 100 °C for two hours.

It should be noted that throughout the entire thin‐film and final PDMS coating process, great care and attention was taken to ensure that all microchip samples were handled properly. To avoid introducing contaminants (such as hand oils or residues) and damage to the thin‐film coatings, the samples were never touched directly. Instead, appropriate tools (tweezers or vacuum pick‐up tools) were employed for handling. Additionally, the packaging and containers used for storage and transport were also maintained in a pristine condition to prevent any contamination. This meticulous approach was essential, as cleanliness is critical for achieving optimal adhesion of the coating layers, which directly influences the reliability and performance of the encapsulation.

### Animals, Implantation, and Explanation Procedures

All in vivo experiments were performed per the EC Council Directive of September 22, 2010 (2010/63/EU). All procedures were reviewed and approved by the Animal Care Committee of the Research Centre for Natural Sciences and the National Food Chain Safety Office of Hungary (license number: PE/EA/1253‐8/2019). Implantation procedures were carried out as follows. Wistar rats (*n* = 6; weight, 300 ± 50 g at the initiation of the study) were anesthetized by an intraperitoneal injection of a mixture of ketamine (75 mg kg^−1^ of body weight; CP‐Ketamin, Produlab Pharma B. V., Raamsdonksveer, The Netherlands) and xylazine (10 mg kg^−1^ of body weight; CP‐Xylazin, Produlab Pharma B. V., Raamsdonksveer, The Netherlands). A body temperature of 37 °C was sustained with an electric heating pad connected to a temperature controller (Supertech, Pécs, Hungary). Before implantation, samples were sterilized by immersing them in isopropyl alcohol for at least 20 min and then washing them with a continuous stream of distilled water for 2 min. To reduce the number of animals used, each animal was subcutaneously implanted with two microchips (see Table S1 Supporting Information) The incision was closed using interrupted sutures followed by a standard combined postoperative analgesic regimen. Samples were explanted at three time points: months 2, 4, or 7. Before explantations, rats were initially anesthetized in the same way as described above. After anesthesia induction, the animals were overdosed with isoflurane (5% in 100% oxygen) until breathing stopped. Before explantations, the skin around the implants was examined for any inflammation. After long‐term implantation, a tissue pocket was formed around each sample. The samples were removed from the pocket to analyze the tissue pocket. The pocket was immersed in 4% paraformaldehyde solution for 24 h, then washed in 0.1 m PBS and stored in PBS. The tissue samples were then sectioned and stained with hematoxylin and eosin stain, and photomicrographs were taken for tissue analysis. Explanted microchips were immediately rinsed with deionized (DI) water, blow‐dried, and stored in a dried condition for a week before analysis. Before analysis, all samples (explanted and reference) were cleaned using a terg‐a‐zyme cleaning detergent prepared according to the manufacturer's specifications (Alconox, Inc.), and thoroughly soaked and rinsed with deionized water.

### Multilayer Stack Characterization Using Scanning Electron Microscopy (SEM)

For realizing all cross sections and scanning electron microscopy (SEM) images, a dual‐beam, focused ion beam (FIB) system (FEI Helios NanoLab600i) was used. A gallium (Ga) ion beam with nanoscale precision was used for milling, and a field emission gun for high‐resolution imaging. Before loading the samples into the SEM system, samples were covered in a sputter coater with a thin conductive layer of platinum. After selecting the area of interest in the FIB system, a 1 µm thick platinum strip was deposited with an in situ gas injector system to protect the surface structure from ion beam‐induced damage. Cross sections were prepared at a tilt angle of 52° and a beam current between 21 and 2.5 nA at 30 kV for coarse and fine milling, respectively. The corresponding SEM images were generated with a through‐the‐lens detector (TLD) using back‐scattered electrons (BSE).

### Surface Characterization Using Atomic Force Microscopy (AFM)

The Bruker Icon was used in tapping and PeakForce Quantitative Nanomechanics (QNM) mode. Per sample, two areas were scanned with an area of 10 µm x 10 µm.

### Chemical Composition and Barrier Property: Time‐of‐flight Mass Spectrometry (ToF‐SIMS) Depth Profiling

ToF‐SIMS analyses were performed using an ION TOF IV instrument at Eurofins EAG Laboratories, Eindhoven, The Netherlands. The instrument was operated in positive and negative mode using 1 keV O^2+^ and 1 keV Cs^+^ sputtering ions, respectively. At negative mode, sputtering was carried out using the Cs^+^ primary ion beam to enhance the detection level of the electronegative species [O^−^] and [OH^−^]. The sputtered area was 250 × 250 µm^2^ and the analyzed area was 50 × 50 µm^2^ centered in the sputtered area. The analysis was done with a beam of 25 keV Bi^+^ ions. To increase the sensitivity for lighter elements, all samples were stored in the ToF‐SIMS instrument under ultra‐high vacuum (<10^−9^ bar) for 64 h before analysis.

## Conflict of Interest

The authors declare no conflict of interest.

## Author Contributions

K.N. planned and designed the experiments, prepared the samples, performed the study, collected and analyzed the data, and wrote and revised the manuscript. M.v.G. and R.R. conducted the thinfilm encapsulation. D.H., K.T., D.M., and I.U. conducted the animal studies and collected and analyzed the data. A.G. conducted the SEM analysis and collected and analyzed the data. I.U., and V.G. supervised the study, acquired the funding, and provided resources. All authors contributed to reviewing and editing the manuscript.

## Supporting information



Supporting Information

## Data Availability

The data that support the findings of this study are available from the corresponding author upon reasonable request.
