# Supplementary material for: An In Vivo Biostability Evaluation of ALD and Parylene‐ALD Multilayers as Micro‐Packaging Solutions for Small Single‐Chip Implants
Source: Small. 2025 Jan 23;21(16):2410141. doi: 10.1002/smll.202410141 (PMC12019904; doi:10.1002/smll.202410141)
Supplement: Supplementary file 1 — Supporting Information [file SMLL-21-2410141-s001.pdf]

NANO · MICRO
small

Supporting Information

for *Small*, DOI 10.1002/smll.202410141

An In Vivo Biostability Evaluation of ALD and Parylene-ALD Multilayers as Micro-Packaging Solutions for Small Single-Chip Implants

*Kambiz Nanbakhsh, Matthias Van Gompel, Riina Ritasalo, Astrid Gollhardt, Domonkos Horváth, Kinga Tóth, Domokos Meszéna, István Ulbert, Wouter Serdijn and Vasiliki Giagka**

Supporting Information

An in vivo Biostability Evaluation of ALD and Parylene-ALD Multilayers as Micro-packaging Solutions for Small Single-Chip Implants

Kambiz Nanbakhsh[1], Matthias Van Gompel[2], Riina Ritasalo[3], Astrid Gollhardt[4], Domonkos Horváth[5,6], Kinga Tóth[5], Domokos Meszéna[5,6], István Ulber[5,6,7], Wouter Serdijn[1,8], Vasiliki Giagka[1,9]*

*Corresponding author: Dr. Vasiliki Giagka, v.giagka@tudelft.nl

[1]Department of Microelectronics, Faculty of Electrical Engineering, Mathematics and Computer Science, Delft University of Technology, Delft, The Netherlands.

[2]Comelec SA, 2301 La Chaux-de-Fonds, Switzerland.

[3]Applied Materials, Finland, Masalantie 365, 02430 Masala, Finland.

[4]Department of Environmental & Reliability Engineering, Fraunhofer Institute for Reliability and Microintregration IZM, 13355 Berlin, Germany.

[5]Research Centre for Natural Sciences, Institute of Cognitive Neuroscience and Psychology, HUN-REN, 1117 Budapest, Hungary.

[6]Pazmany Peter Catholic University, Faculty of Information Technology and Bionics, 1083 Budapest, Hungary.

[7]Department of Neurosurgery and Neurointervention, Faculty of Medicine, Semmelweis University, Amerikai út 57, 1145 Budapest, Hungary.

[8]Department of Neuroscience Erasmus Medical Center, Rotterdam, The Netherlands.

[9]Department of System Integration and Interconnection Technologies, Fraunhofer Institute for Reliability and Microintregration IZM, 13355 Berlin, Germany.

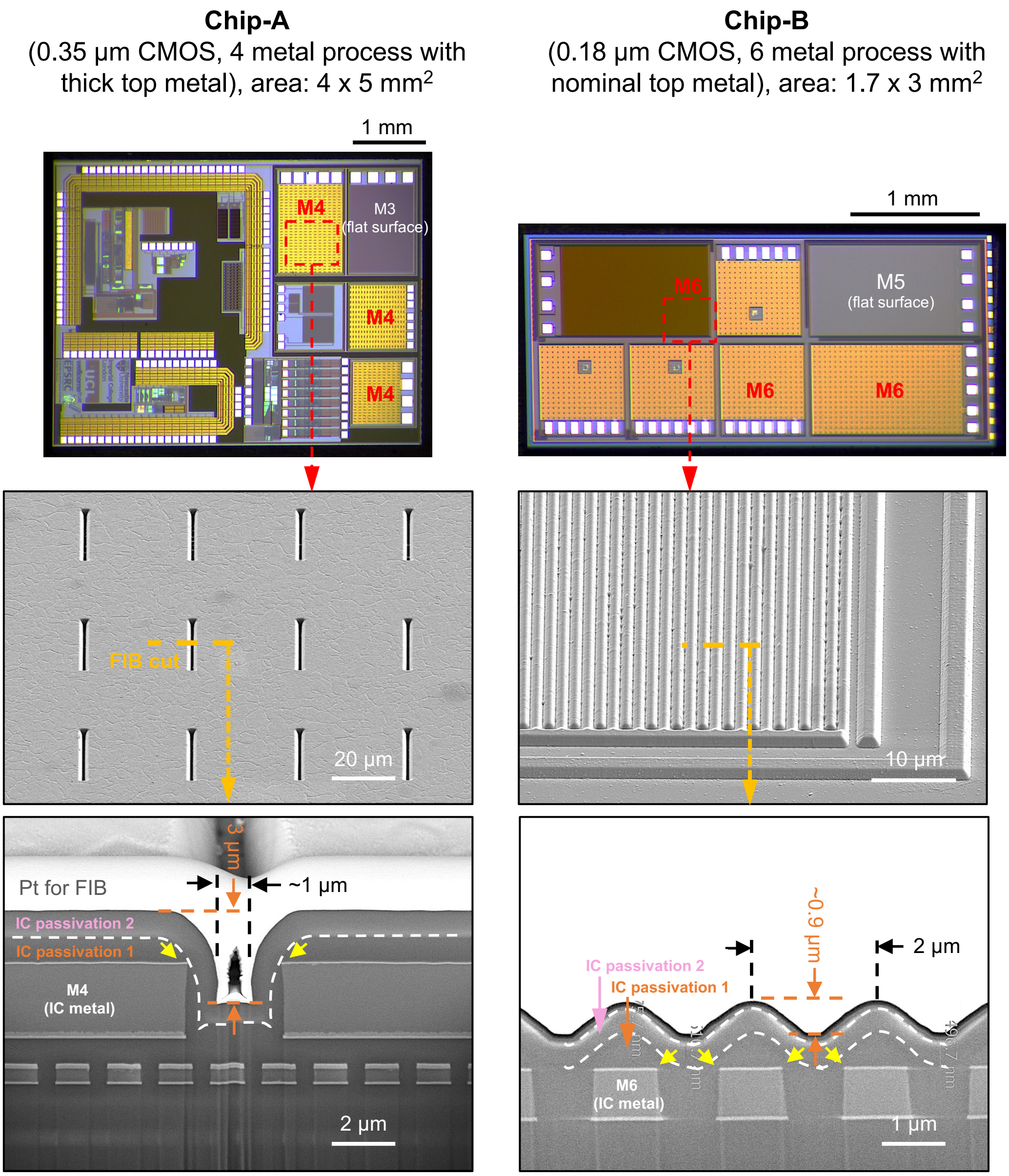

Figure S1. Optical and scanning electron microscope (SEM) images of uncoated Chip-A and Chip-B samples showing the surface microtopography on the chips resulting from using the top metallization of the process. Chip-A manufactured using a 0.35 µm complementary metal-oxide-semiconductor (CMOS) 4-metal process with thick top metal 4 (M4). Chip-B manufactured using a 0.18 µm CMOS 6-metal process with nominal top metal 6 (M6). Both microchips are coated using a dual passivation layer (IC passivation 1 and 2). The use of the top metal creates non-flat surfaces with aspect ratios of 3 µm (height) / 1 µm (width) for Chip-A and 0.9 µm (height) / 2 µm (width) for Chip-B. It is important to note that the use of the top metal results in step coverage, which leads to non-conformality in the IC's own passivation. This causes a thinner 'IC passivation 1' layer near the edges of the metal features (see yellow arrows). Both Chip-A and Chip-B ICs include various structures implemented using the top metallization, designed to achieve the highest possible aspect ratios

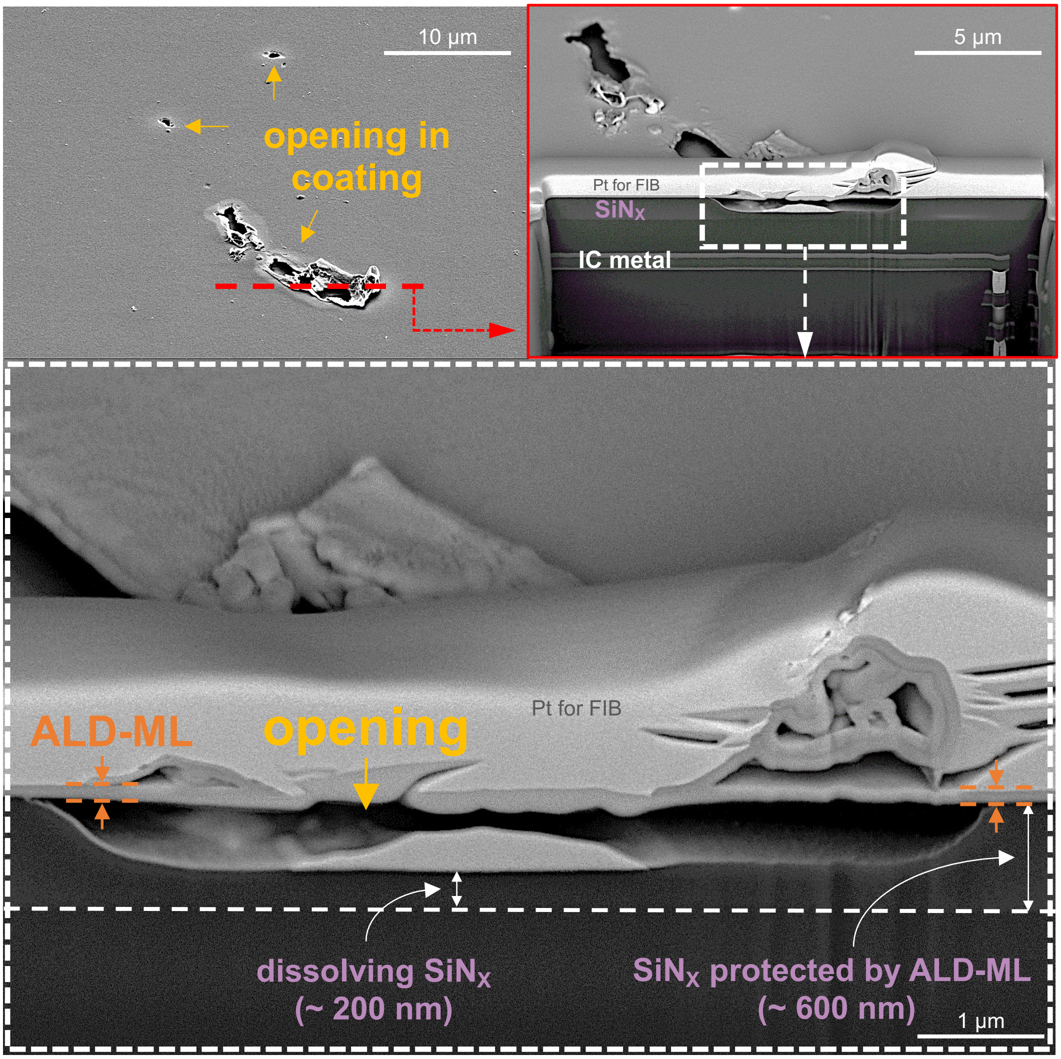

Figure S2. Tilted surface and cross-sectional SEM images of a 7-month explanted Chip-A, coated with the ALD-ML film, showing opening in the ALD and degradation of the IC material. In the top-left image, the dotted line indicates the area used for focused ion beam (FIB) cross-sectioning. Cross-sectional SEM images show degradation and under-etching of the microchip's silicon nitride (SiN_X) passivation due to the opening in the ALD-ML coating, resulting in its direct exposure to tissue environment. After 7 months, ~400 nm of the entire ~600 nm SiN_X has dissolved due to direct exposure to tissue and body fluids.

Table S1. Implantation of coated microchips, explanation time points and analysis.

Animal	Left-side	Right-side	Explanation date	Analysis
#1	Chip-A (ALD-ML)	Chip-B (ParC-ALD-ML)	2-month	Microscopy
#2	Chip-B (ALD-ML)	Chip-A (ParC-ALD-ML)	2-month	
#3	Chip-A (ALD-ML)	Chip-B (ParC-ALD-ML)	4-month	Microscopy/SEM
#4	Chip-B (ALD-ML)	Chip-A (ParC-ALD-ML)	4-month	
#5	Chip-A (ALD-ML)	Chip-B (ParC-ALD-ML)	7-month	Microscopy/AFM /SEM/ ToF-SIMS
#6	Chip-B (ALD-ML)	Chip-A (ParC-ALD-ML)	7-month	

Biocompatibility

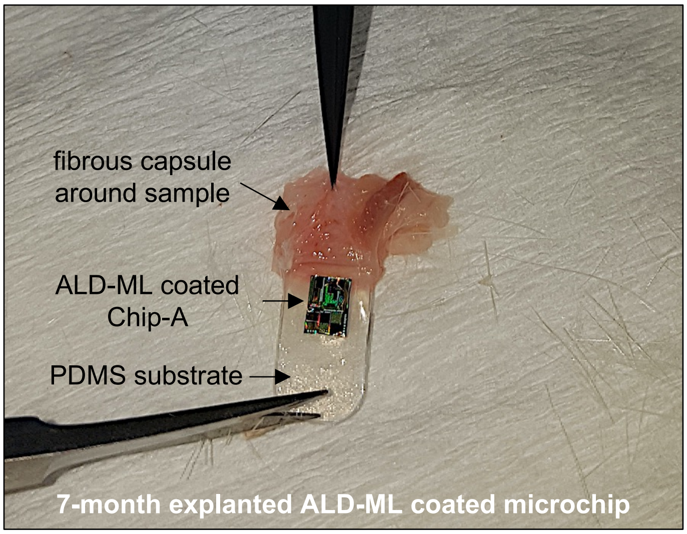

Figure S3. Image of an explanted ALD-ML coated Chip-A after 7 months of implantation. Easy tissue removal is seen on the ALD-ML coated chip.

Before each explanation (at 2, 4 and 7 month), careful observation of the rats' skin after shaving showed perfect healing of the implant incisions. There is no redness of the back and no scabbing or scarring. For explantation, an incision was made next to the implantation incision. The skin and subcutaneous membranes were carefully cut. The samples were contained within a tissue pocket, which consist of a very thin (<0.2 mm), flexible, well-vascularized and strong membrane. Once the tissue pocket was cut open, the samples came out without any resistance. All samples (HfO_2-based ALD-ML and ParC-ALD-ML) had no adhering tissue on the surface and came out in pristine condition without additional damage to the tissue.

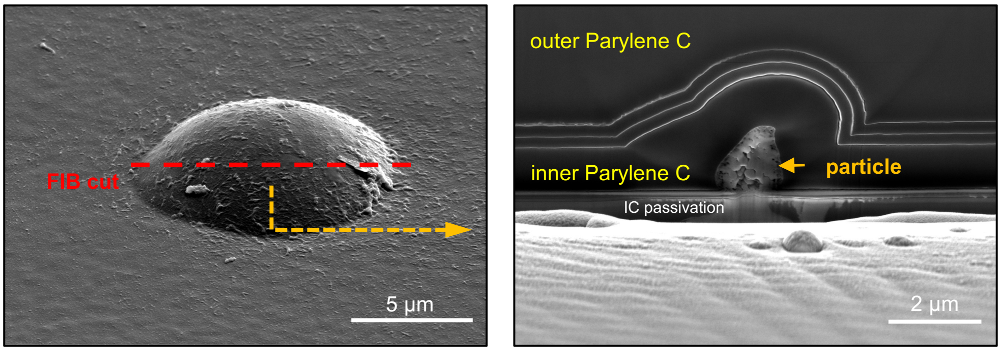

Figure S4. Tilted surface and cross-sectional SEM image of a surface particle on Chip-A, coated with the hybrid ParC-ALD-ML showing how the hybrid multilayer can conformally coat a particle ~1 µm in diameter. Red dashed line shows the region used for the focused ion beam (FIB) cut.

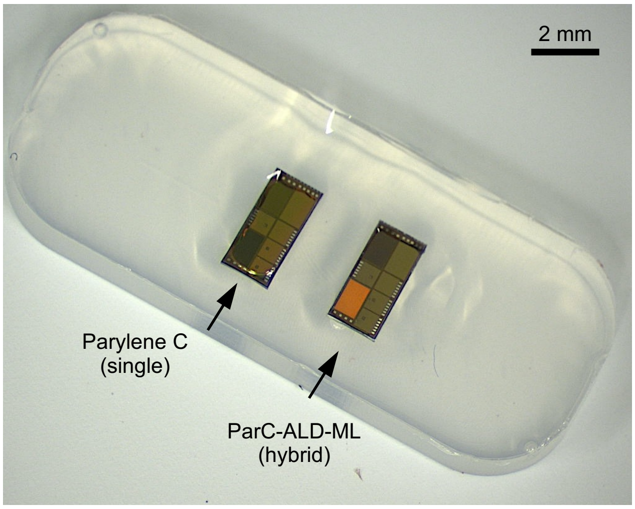

Figure S5. A representative image of two Chip-B ICs coated with a single and hybrid Parylene C multilayer (ParC-ALD-ML) explanted after 7 months. To compare the mechanical integrity of the hybrid multilayer to a single layer a group of ICs, Chip-A (n=2) and Chip-B (n=2), were coated using a single ~4 µm Parylene C layer. Prior to coating, an initial O_2 plasma treatment step followed by a silane (A174) adhesion promoter step was included in the cycle. Post in vivo aging (at 4 and 7 months) showed no difference between the single and hybrid coating.

Supporting Note 1: After the animal in vivo aging, to qualitatively evaluate adhesion strength between the PDMS and the two coatings under investigation (ALD-ML or ParC-ALD-ML), a shear force was applied to the PDMS edge (see figure below). This was performed using a pair of plastic tip tweezers while microscopically inspecting (at 24x magnification) for any detachment between the PDMS and the coating. The adhesion was qualitatively scored as follows:

- **Low**: Delamination between PDMS and coating was already present without the need of shear force.
- **Medium**: PDMS could be peeled off from the coating with minimum force, leaving no residues on the surface.
- **High**: PDMS could not be removed and mainly resulted in the cohesive breakage of PDMS itself.

For all ParC-ALD-ML coated chips, PDMS delamination was already visible on 2-month explanted samples (see red arrow on figure below). For the ALD-ML coated chips, PDMS adhesion was scored high given that the shear force always resulted in cohesive breakage of PDMS itself.

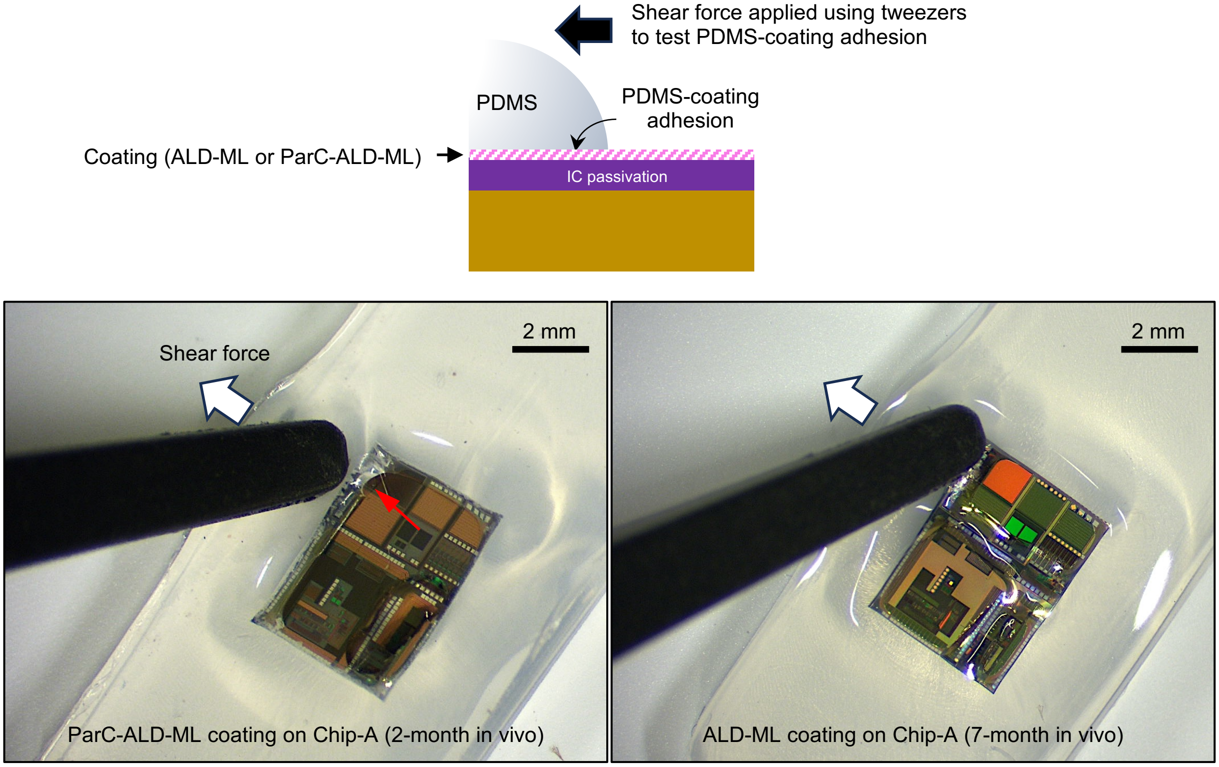

Supporting Note 2: For implantable electronics, the selection of the coating's WVTR depends on two key factors:

1) The moisture sensitivity of the electronic materials and circuitry. The selection of the coating's WVTR greatly depends on the moisture-sensitivity level of the components underneath. For components highly sensitive to moisture (such as copper used as metal traces [1] or capacitors with porous dielectrics [2]), a coating with a lower WVTR is required to ensure device reliability. With regards to ICs, the moisture sensitivity depends on the fabrication materials, which vary by foundry and technology node. Studies have shown that newer IC nodes (<90 nm CMOS) are more susceptible to moisture due to the use of porous dielectric films in their material stack [3-4]. For these ICs, a low-WVTR coating is essential, particularly when targeting long operational lifetimes.

The choice of coating is also influenced by the electrical signals and how often the device is powered. For example, as shown in [5], a microsystem coated with Parylene C/ALD exhibited a ~13-fold longer operational lifetime when it was left unbiased and only routinely monitored, compared to when it was continuously powered. This difference arises primarily from the role of electrical signals in driving electrochemical reactions once moisture permeates the coating and reaches the underlying components and interfaces [1, 6]. Consequently, for devices requiring frequent use and continuous powering, a coating with a lower WVTR can significantly enhance lifetime reliability.

If the electronic materials underneath are inherently moisture-resistant and non-corrosive, a single Parylene C layer may be sufficient. However, determining the moisture sensitivity of electronic components often requires detailed knowledge of their manufacturing process, which is not always readily available. As a result, hybrid coatings with a lower WVTR are a safer and more reliable option, especially when cost and process complexity are not significant constraints. For embedded systems with more device complexities (more components and exposed signal-carrying metals), using a low WVTR coating can be considered a more reliable encapsulation solution.

2) The intended operational lifetime of the device. Besides the moisture sensitivity level of the electronic components underneath, the targeted operation lifetime of the device greatly drives the selection of the coating's WVTR. For example, for short-term applications (1-2 months) in vivo, a single Parylene C layer would suffice, irrespective of the electronics' moisture sensitivity. For longer-term applications (>1 year), a coating with lower WVTR would delay moisture permeation and increase the lifetime reliability of the device.

Supporting Note 3: With regard to future applications, for embedded devices and systems which include multiple rigid off-the-shelf components (e.g. microcontrollers, capacitors, resistors and inductors) considerations should be taken to achieve a successful encapsulation. The three main considerations are spacing, standoff and cleanliness, which are explained below.

Spacing: Generally, compared to ICs, off-chip components like capacitors and resistors have larger spacings between their signal-carrying connecting terminals. For example, for small 0201 surface mount capacitors, the spacing between the connecting terminals is ~450 μm, while the spacing between the IC pads is usually < 100 μm. Therefore, for this aspect, we believe that off-chip components will be less risky.

Standoff: For achieving conformality on assembled components, sufficient standoff (spacing between the component body and the substrate) should be provided to ensure sufficient penetration of the coating gases and optimal conformality. In the case of a wire-bonded chip, standoff is not a risk as the thick silicon bulk (~200-300 μm) is relatively hermetic itself and all the sensitive functionally is positioned in the top 10 μm of the chip. For flip-chip assembled ICs using ball grid arrays (BGA), however, standoff is critical as the active area of the die is facing the substrate and attention should be given with respect to the standoff/chip dimension aspect ratio.

Cleanliness and adhesion: Cleanliness of the surface has a significant effect on the adhesion and encapsulation performance [1], [7]. Silicon-ICs are generally clean as all of the fabrication and processing is done in dedicated clean-rooms. For embedded systems that are assembled using surface mount technology and soldering, dedicated steps are needed to ensure a flux-free surface after the assembly process. It should be noted that, in case of surface contamination, even if adhesion between the coating and electronics is compromised, using a low WVTR coating can delay moisture permeation and leakage-induced failures.

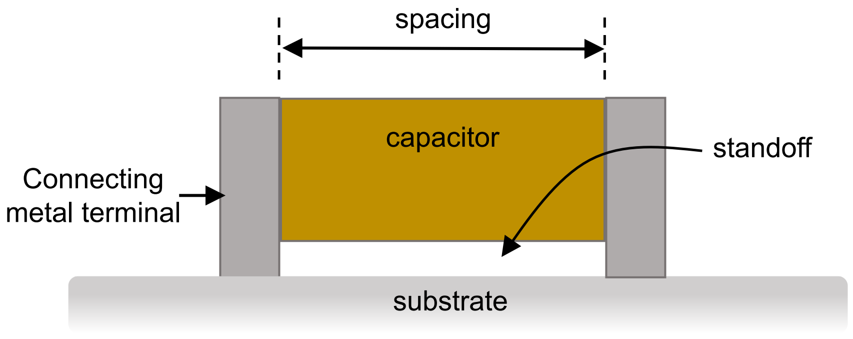

References:

[1] Baylakoğlu İ, Fortier A, Kyeong S, Ambat R, Conseil-Gudla H, Azarian MH, Pecht MG. The detrimental effects of water on electronic devices. e-Prime-Advances in Electrical Engineering, Electronics and Energy. 2021 Jan 1;1:100016.

[2] Lehmann V, Hönlein W, Reisinger H, Spitzer A, Wendt H, Willer J. A novel capacitor technology based on porous silicon. Thin Solid Films. 1996 Apr 15;276(1-2):138-42.

[3] Michelon J, Hoofman RJ. Moisture influence on porous low-k reliability. IEEE Transactions on Device and Materials Reliability. 2006 Jun;6(2):169-74.

[4] Mischler L, Cartailler V, Imbert G, Duchamp G, Frémont H. Investigating the degradation mechanisms of moisture on the reliability of integrated low-k stack. Microelectronics Reliability. 2023 Nov 1;150:115087.

[5] Xie X, Rieth L, Caldwell R, Negi S, Bhandari R, Sharma R, Tathireddy P, Solzbacher F. Effect of bias voltage and temperature on lifetime of wireless neural interfaces with Al 2 O 3 and parylene bilayer encapsulation. Biomedical microdevices. 2015 Feb;17:1-8.

[6] Nanbakhsh K, Kluba M, Pahl B, Bourgeois F, Dekker R, Serdijn W, Giagka V. Effect of signals on the encapsulation performance of parylene coated platinum tracks for active medical implants. In2019 41st Annual International Conference of the IEEE Engineering in Medicine and Biology Society (EMBC) 2019 Jul 23 (pp. 3840-3844). IEEE.

[7] Borges CS, Marques EA, Carbas RJ, Ueffing C, Weißgraeber P, Silva LD. Review on the effect of moisture and contamination on the interfacial properties of adhesive joints. Proceedings of the Institution of Mechanical Engineers, Part C: Journal of Mechanical Engineering Science. 2021 Feb;235(3):527-49.
